# ANORECTAL MANOMETRY STANDARD OF A BRAZILIAN POPULATION AT PRODUCTIVE AGE WITHOUT PELVIC FLOOR DISORDERS: A PROSPECTIVE VOLUNTEERED STUDY

**DOI:** 10.1590/0102-672020210001e1580

**Published:** 2021-06-11

**Authors:** Rodrigo Ambar PINTO, Isaac José Felippe CORREA-NETO, Leonardo Alfonso BUSTAMANTE-LOPEZ, Caio Sergio R. NAHAS, Carlos Frederico S. MARQUES, Carlos Walter SOBRADO-JUNIOR, Ivan CECCONELLO, Sergio Carlos NAHAS

**Affiliations:** 1 Hospital das Clínicas, Discipline of Coloproctology, Faculty of Medicine, University of São Paulo, São Paulo, SP, Brazil

**Keywords:** Manometry, Anal canal, Evaluation study, Pelvic floor, Manometria, Canal anal, Avaliação da deficiência, Distúrbios do assoalho pélvico

## Abstract

**Background:**

Due to the lack of normal standards of anorectal manometry in Brazil, data used are subject to normality patterns described at different nationalities.

**Aim:**

To determine the values and range of the parameters evaluated at anorectal manometry in people, at productive age, without pelvic floor disorders comparing the parameters obtained between male and female.

**Methods:**

Prospective analysis of clinical data, such as gender, age, race, body mass index (BMI) and anorectal manometry, of volunteers from a Brazilian university reference in pelvic floor disorders.

**Results:**

Forty patients were included, with a mean age of 45.5 years in males and 37.2 females (p=0.43). According to male and female, respectively in mmHg, resting pressures were similar (78.28 vs. 63.51, p=0.40); squeeze pressures (153.89 vs. 79.78, p=0.007) and total squeeze pressures (231.27 vs. 145.63, p=0.002). Men presented significantly higher values of anorectal squeeze pressures, as well as the average length of the functional anal canal (2.85 cm in male vs. 2.45 cm in female, p=0.003).

**Conclusions:**

Normal sphincter pressure levels in Brazilians differ from those used until now as normal literature standards. Male gender has higher external anal sphincter tonus as compared to female, in addition a greater extension of the functional anal canal

## RESUMO

**Racional:**

Devido à falta de padrões normais de manometria anorretal no Brasil, os dados utilizados estão sujeitos a padrões de normalidade descritos em diferentes nacionalidades .

**Objetivo:**

Determinar os valores e a faixa da manometria anorretal de pessoas em idade produtiva, sem distúrbios do assoalho pélvico, comparando os parâmetros obtidos entre homens e mulheres.

**Métodos:**

Análise prospectiva de dados clínicos, como gênero, idade, raça, índice de massa corporal (IMC) e manometria anorretal, de voluntários de uma referência universitária brasileira em distúrbios do assoalho pélvico.

**Resultados:**

Quarenta pessoas foram incluídas, com idade média de 45,5 anos nos homens e 37,2 nas mulheres (p=0,43). De acordo com homens e mulheres, respectivamente em mmHg, as pressões de repouso foram semelhantes (78,28 vs. 63,51, p=0,40); pressões de contração (153,89 vs. 79,78, p=0,007) e pressão total de compressão (231,27 vs. 145,63, p=0,002). Os homens apresentaram valores significativamente maiores de contração esfincteriana, assim como o comprimento médio do canal anal funcional (2,85 cm nos homens vs. 2,45 cm nas mulheres, p=0,003).

**Conclusões:**

Os níveis normais de pressão esfincteriana no Brasil diferem dos utilizados até o momento como padrão normal da literatura. O gênero masculino apresenta maior tônus ​​do esfíncter anal externo em relação ao feminino, além de maior extensão do canal anal funcional

## INTRODUCTION

Anorectal manometry is one of the most widely used and studied physiological tests for the evaluation of patients with pelvic floor disorders, specially continence disorders, whether they have anal incontinence or constipation. It is an important examination and used in research centers, clinics or specialized hospitals searching for anorectal disorders^22^ ^,^ ^24^; it may suggest the diagnosis and guide the management^15^ ^,^ ^22^ ^,^ ^24^. This anorectal physiology test has a well-established impact in the evaluation of the abnormalities of the anorectal sphincter function and anal coordination during defecation^1^ ^,^ ^7^ ^,^ ^10^ ^,^ ^18^ ^,^ ^23^.

Anorectal manometry can objectively be focused on the following data: resting and squeeze pressures, functional anal canal length, coordination of muscle relaxation during defecation, as well as the ability to sustain the sphincter contraction, the recto anal inhibitory reflex, rectal sensitivity (minimum volume to induce sensation of evacuation) and maximum rectal capacity (or maximum tolerable volume) ^1,3,10,18^.

Due to the lack of studies that demonstrate normal anorectal manometry parameters in our country, the data found in the Brazilian population are subject to normality patterns described by studies of different nationalities^18^ ^,^ ^22^. This phenomenon might carry imprecise information to our patients.

 Approximately 60% of patients with symptoms of anal incontinence may present normal manometric values^16^ ^,^ ^19^. Anorectal manometry clinical usefulness may be limited by the variation of types of devices used in the market, due to a deficiency of standardized protocols in different facilities and what are considered the normal parameters in healthy individuals without pelvic floor disorders^7^ ^,^ ^13^ ^,^ ^18^.

This research aimed to determine anorectal manometry values and range in patients, at productive age, without pelvic floor disorders, previous anorectal surgeries or parity, comparing the parameters obtained between the male and female.

## METHODS

This study was approved by institutional ethics committee under n^o^. 513190. It is a prospective analysis of clinical data, such as gender, age, race, body mass index (BMI) and anorectal manometry, of volunteers from the outpatient Department of Digestive System Surgery, Hospital das Clínicas, Medical School, University of São Paulo, São Paulo, SP, Brazil.

From October 2015 to January 2018, persons of both genders with BMI between 18.5 and 29.9 kg/m^2^, without known pelvic floor disorders and women without obstetrical history, with normal anal continence and without any ROME III^13^ criteria for constipation were included in the study. Non-inclusion criteria were patients with diabetes mellitus or who did not consent to perform anorectal manometry or participation.

The preparation for anorectal manometry consisted of an evacuation enema at least 2 h before the exam. Volunteers were placed in the left lateral decubitus position (Simms position) with the lower limbs semi-flexed and the head resting on a pillow in a quiet and comfortable environment. The technique used for the anorectal manometry was stationary, in which the catheter was inserted up to 6 cm from the anal merge and traction distally every centimeter to zero.

Anorectal manometry was performed using a Multiplex 2^®^ fluid perfusion manometer, respective software and anorectal catheter with eight radial channels and one distal channel coupled to a balloon (Alacer Biomédica^®^). The values of all the following parameters were obtained: mean rest pressure, functional anal canal length, mean external anal sphincter contraction pressures and total voluntary contraction in the functional anal canal, retoanal inhibitory reflex, sensitivity (minimum volume to induce sensation of evacuation) and the capacity (maximum tolerable volume) of the rectum according to gender, race and age group to obtain limits and to observe this sample population standard.

### Statistical analysis

Variables descriptive analysis was performed. Quantitative ones were presented in terms of their central (mean) and dispersion (standard deviation and error) values. We adopted as values within normality the average plus or minus 2 standard errors. To compare the male and female, the t test was used and the level of significance was 5%. The statistical software used was SPSS 22.0 for Windows (IBM^®^).

## RESULTS

Twenty men and 20 women were included in the study, following the inclusion and exclusion criteria. The mean age was 45.5±10.73 years in male and 37.2±9.11 years in female (p=0.43). The mean BMI was 25.46±3.66, been 25.48 for men and 24.43 for women. Regarding race, 90% were white, 5% black and 5% mixed race. The mean values found in the studied population of anorectal manometric parameters are shown in Table 1.

**TABLE 1:** Parameters of anorectal manometry in the study population

	Average	Minimum	Maximum	Standard deviation
Resting pressure (mmHg)	70.89	62.52	79.26	21.78
Total contraction pressure (mmHg)	188.45	160.87	216.02	71.72
External anal sphincter contraction pressure (mmHg)	116.83	91.26	142.4	66.53
Functional anal canal (cm)	2.65	2.30	2.99	0.89
Sustaining capacity (%)	80.1	73.91	86.24	16.03
Rectal sensitivity (ml)	58.5	39.91	77.08	48.36
Rectal capacity (ml)	156.87	129.11	184.63	72.21

### Sphincter pressure

**FIGURE 1:**
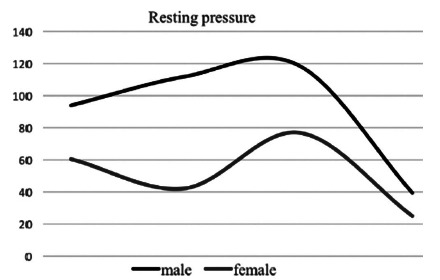
Resting anal pressure according to gender

**FIGURE 2:**
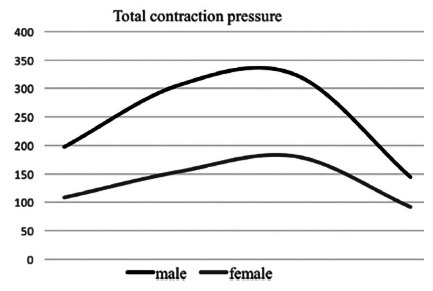
Anal pressure of total voluntary contraction according to gender

When comparing the genders, it was verified that, according to Table 2, the values of the resting pressures and voluntary contraction are lower in females, with statistical significance for the external anal sphincter pressures alone and total voluntary contraction pressures. Resting pressures, internal anal sphincter, and lift capacities did not show significant difference between the genders. Figures 1 and 2 show differences in sphincter pressure according to gender.

### Functional anal canal

Analyzing the length of the functional anal canal between genders, this was significantly lower in women compared to men (Table 2). The length of the functional anal canal was closer to 2 cm in women and 3 cm in men.

### Rectal sensitivity and capacity

The mean rectal sensitivity and capacity did not present statistically significant differences between the genders (Table 2). There was a tendency to less rectal sensitivity in men.

**TABLE 2:** Comparison of anorectal manometry data between male and female

	Male (average, min., max.)	Female (average, min., max.)	p
Resting pressure	78.28 (39.4-118.7)	63.51 (24.9-98.5)	0.406
External anal sphincter contraction pressure	153.89 (59.2-307)	79.78(22.3-135.7)	0.007
Total contraction pressure	231.27 (133-335.1)	145.63 (88.2-211.5)	0.002
Length of functional anal canal (cm)	2.85 (2-4)	2.45 (1-5)	0.003
Lifting capacity (%)	78.75 (48.6%-101%)	81.64 (51.8-104.1%)	0.298
Rectal sensitivity (ml)	70.25 (15-280)	46.75 (10-100)	0.082
Rectal capacity (ml)	181.75 (85-360)	132.00 (60-255)	0.211

## DISCUSSION

The performance of standardized studies in the Brazilian population, either by the selection and exclusion of patients, or by the technique used to perform anorectal manometry, is scarce in our country. Therefore, the Brazilian population adopt reference parameters established in populations quite different from the Brazilians, having no reference of patients without pelvic floor disorders.

Analyzing the anorectal manometry data in healthy Brazilian volunteers, without pelvic floor disorders, fecal incontinence or constipation, obesity, obstetric history and previous history of orthopedic and/or colorectal surgeries, it can be verified that the parameters obtained were, in general, different from those considered the international standard by world literature^4^ ^,^ ^8^ ^,^ ^11^ ^,^ ^17^, and when comparing the genders, data are even more discrepant.

Morgado et al^17^ in a study involving 466 patients with no exclusion criteria for risk for pelvic floor disorders, demonstrated mean resting pressure of 56.26 mmHg and contraction pressure of the external anal sphincter of 81.25 mmHg. In the present study, the mean resting pressures and contraction of the external anal sphincter in a controlled population were 70.89 and 116.83 mmHg, respectively.

Lombardo et al^13^ conducted a study very similar to the present one, evaluating the parameters of anorectal manometry in 52 healthy people, of which 22 were nulliparous women. They also demonstrated a significant reduction in the contraction pressure in women compared to men, and a lower resting pressure, but without statistical significance. On the other hand, a similarity was observed in the length of the functional anal canal, different from the present study, in which there was a greater length of the functional anal canal in men with statistical significance. Evaluating the limits of resting pressures (60-93 mmHg) and voluntary contraction (138-279 mmHg) in that study, we observe that these limits are also higher than previously reported in the literature^8^ ^,^ ^11^ ^,^ ^17^. Sphincter pressure parameters taken as standard in Brazil to date are based on North American studies, range from 40-70 mmHg for resting pressures and from 100-180 mmHg for total voluntary contraction. Normal rectal sensitivity ranges from 10-40 ml and rectal capacity from 100-300 ml^6^ ^,^ ^11^.

Lee et al^12^ analyzed 54 healthy subjects without complaints of pelvic floor disorders by performing high resolution anorectal manometry and showed significantly lower mean resting pressure in females than in males (32 vs. 46 mmHg, p<0.001), as well as at contraction pressures (75 vs. 178 mmHg, p<0.001). Still, rectal sensitivity was similar between the genders (p=0.855). These data support that high resolution anorectal manometry information is similar to conventional manometry.

Likewise, in the present study, mean total contraction pressures (231.27 vs. 145.63 mmHg, p=0.002) and isolated external anal sphincter pressures (153.89 vs. 79.78, p=0.007) were significantly higher in men when compared to women. Among other factors, the greater density of muscle mass and contractile force in male individuals could justify the higher contraction pressures^5^.

In a recent study, Carrington et al^3^ carried out a survey of 107 physicians with conventional or high-resolution anorectal manometry in 30 countries, not including Brazil. Conventional manometry was used in 47% of the centers. It has been shown that 74% of establishments perform more than two manometries a week and only 8% perform more than 20 tests per week. In the present study, the exams were performed in a single reference center in the anorectal functional evaluation, having an average of 10 exams per week.

Regarding the significant variety of the anorectal manometry test, standardization of technique and results in that same study^3^, only 29% of the interviewed mentioned mean resting pressure at the level of the functional anal canal. When analyzing of voluntary contraction, there was an equivalence in the responses regarding the use of the total contraction or only the increase in the resting pressure. Also, the sphincter pressure measurement method presented 18 possible ways among the physicians who answered the survey. Regarding the measurement of sustained contraction pressure, the variation of the methodology of the interviewed was even greater with 43 ways of obtaining the data. In addition, only 44.9% of the centers studied undergo the rectal sensitivity test.

The evaluation of protocols and technical analyzes in different centers of different nationalities shows numerous disparities, which could make even more complicated the use of an anorectal manometric standard established in one country, based on data from other countries. Therefore, in addition to the population limitation, which differs from one nationality to another, there is also a technical limitation to the examination, depending on the protocol adopted by each institution and even on the brand and generation of the equipment used.

Based on data collected in the present study, it is verified that the information obtained on anorectal manometry in this sample of the Brazilian population analyzed in a large national institution may explain cases previously considered incontinent in the clinic, but with pressure indexes previously presented as normal, based on data of literature. In addition, the population was controlled to include only non-elderly and non-obese patients, which could influence manometric values^19^ and the similar age between genders eliminates a possible confounding factor that could influence the results.

It should also be noted that, similarly to the literature^3^ ^,^ ^9^ ^,^ ^20^ ^,^ ^25^, in colorectal physiology evaluation, even through objective data of anorectal manometry, there is a significant complexity in the interpretation, standardization of the technique and establishment of normality parameters in the examination, considering the multifactorial nature of pelvic floor disorders and variation of populations and groups studied^2^ ^,^ ^21^ ^,^ ^25^ ^,^ ^26^.

Among the limitations of the study, we can mention the restriction of the sample, and the use of conventional anorectal manometry apparatus, in detriment of the high resolution. However, it should be emphasized the difficulty of recruiting volunteers for the study and the greater availability of conventional anorectal manometry devices nowadays in Brazil. In addition, the analysis was performed in a homogeneous group of healthy patients, with similar age and body mass index in a single specialized service with high volume of exams.

## CONCLUSION

The pressure levels found in a group of controlled people and without pelvic floor disorders are different from those used until then as standards of normality of the literature applied to patients throughout the country. The national standards of anorectal manometry need to be modified and adapted to our reality, so new limits of normality like those obtained in this study are suggested. Male have higher external anal sphincter tonus as compared to female, in addition to the greater extension of the functional anal canal.
